# Cell fiber-based 3D tissue array for drug response assay

**DOI:** 10.1038/s41598-022-11670-2

**Published:** 2022-05-12

**Authors:** Midori Kato-Negishi, Jun Sawayama, Masahiro Kawahara, Shoji Takeuchi

**Affiliations:** 1 Department of Bio-Analytical Chemistry, Research Institute of Pharmaceutical Sciences, Faculty of Pharmacy, Musashino University, 1-1-20 Shinmachi, Nishitokyo, Tokyo, 202-8585 Japan; 2 Institute of Industrial Science, The University of Tokyo, 4-6-1 Komaba, Meguro-ku, Tokyo, 153-8505 Japan; 3 Department of Mechano-Informatics, Graduate School of Information Science and Technology, The University of Tokyo, 7-3-1 Hongo, Bunkyo-ku, Tokyo, 113-8656 Japan; 4 International Research Center for Neurointelligence (WPI-IRCN), The University of Tokyo Institutes for Advanced Study (UTIAS), The University of Tokyo, 7-3-1 Hongo, Bunkyo-ku, Tokyo, 113-8656 Japan

**Keywords:** Engineering, Biomedical engineering

## Abstract

For the establishment of a reproducible and sensitive assay system for three-dimensional (3D) tissue-based drug screening, it is essential to develop 3D tissue arrays with uniform shapes and high cell numbers that prevent cell death in the center of the tissue. In recent years, 3D tissue arrays based on spheroids have attracted increased attention. However, they have only been used in specific tissues with hypoxic regions, such as cancer tissues, because nutrient deprivation and hypoxic regions are formed in the core as spheroids grow. Herein, we propose a method to array cell-encapsulated tube-like tissue (cell fiber (CF)) with diameters < 150 μm to prevent nutrient deprivation and hypoxia using a device that can fix the CFs, section them in uniform sizes, and transfer them to a 96-well plate. We fabricated the arrays of CF fragments from cell lines (GT1-7), cancer cells (HeLa), mouse neural stem cells (mNSCs) and differentiated mNSCs, and performed drug response assays. The array of CF fragments assessed the drug response differences among different cell types and drug responses specific to 3D tissues. The array of CF fragments may be used as a versatile drug screening system to detect drug sensitivities in various types of tissues.

## Introduction

Drug screening systems using three-dimensional (3D) tissues have received increased attention in recent years as preclinical tools to understand drug metabolism and toxicity^1–6^. Achievement of reproducible drug screening by using 3D tissue arrays requires cell number uniformity and reduced variations in tissue shape that cause differences in cell viability^7^. Tissue size control is also an important factor for the fabrication of 3D tissue arrays because hypoxia-induced gene expression and cell death, which are usually not observed in healthy tissues in vivo, are caused by increased tissue thickness^8–15^. Spheroids are the most commonly used 3D tissues, and their spherical multicellular aggregates are created in a scaffold-free environment. Given that spheroids can be easily formed on commercially available, ultralow attachment 96-well plates, spheroid arrays have been used for drug screening. However, in some types of cells, the volume and shape of the spheroid dynamically change over the course of the culture, resulting in nonspherical spheroids which cannot easily attain uniform shapes^7,12,16^. Changes in spheroid shape cause differences in the cell viability due to changes in the size of the cell proliferation area^7^. In addition, the number of cells on the surface exposed to the drug changes due to changes in the surface area, as well as penetration into the spheroid. Small spheroids made from fewer than 500 cells tend to have nonuniform sizes^17,18^. Furthermore, if the spheroids grow to attain diameters > 150 μm, hypoxic regions are created inside the tissue^19^. Consequently, the control of the spheroid sizes exerted to match the different growth rates of each cell type is a complex process. Therefore, the development of 3D tissue arrays with minimal variation in cell number and shape, and with simplified size control of tissues in various types of cells, is required.

**Figure 1:**
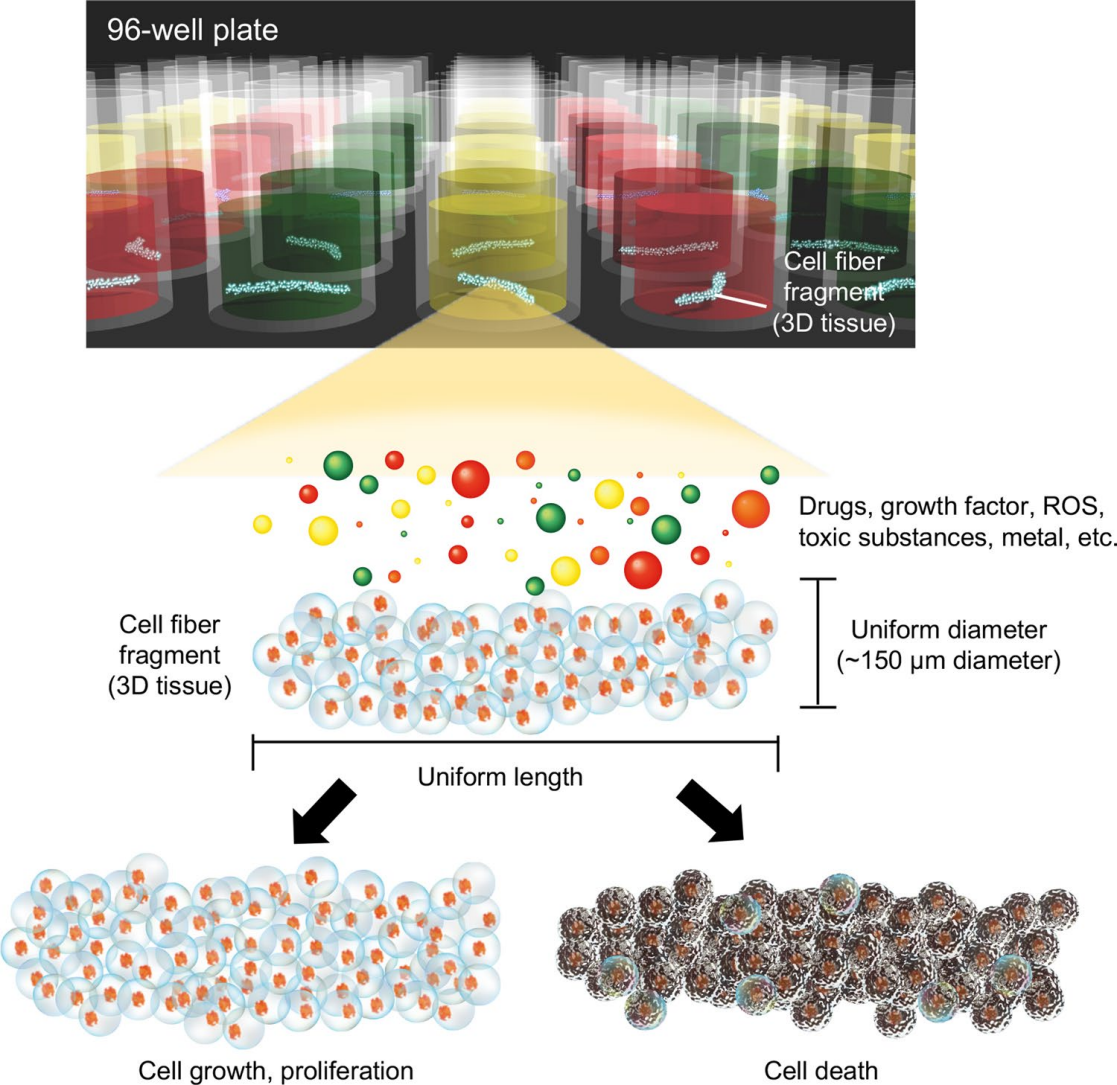
Conceptual illustration of drug screening using an array of the CF fragments on a 96-well plate. We propose an array of the CF fragments formed on a 96-well plate from cell fibers of uniform shape and size as a 3D tissue array for drug screening. Cell viability after drug treatment was detected as a luminescence signal proportional to the adenosine triphosphate (ATP) content of the cells.

This paper proposes a method to create arrays of uniformly sized cell-encapsulated short-tube-like tissue (cell fiber (CF) fragments)^20–22^ (Fig. 1). The CF fragment is < 150 μm in diameter and 5 mm in length and is covered with alginate hydrogel. The proposed array of CF fragments has the following features: (1) the shapes of the CF fragments are maintained uniform because they are covered with alginate hydrogel until drug treatment, and the alginate hydrogel is removed immediately before drug treatment; (2) the cell numbers of the CF fragments are uniform and suitable for applications to bioluminescence assays for cell viability assessments, and (3) the CF fragments of diameters < 150 μm prevents low-nutrient conditions and hypoxia inside the tissues.

**Figure 2:**
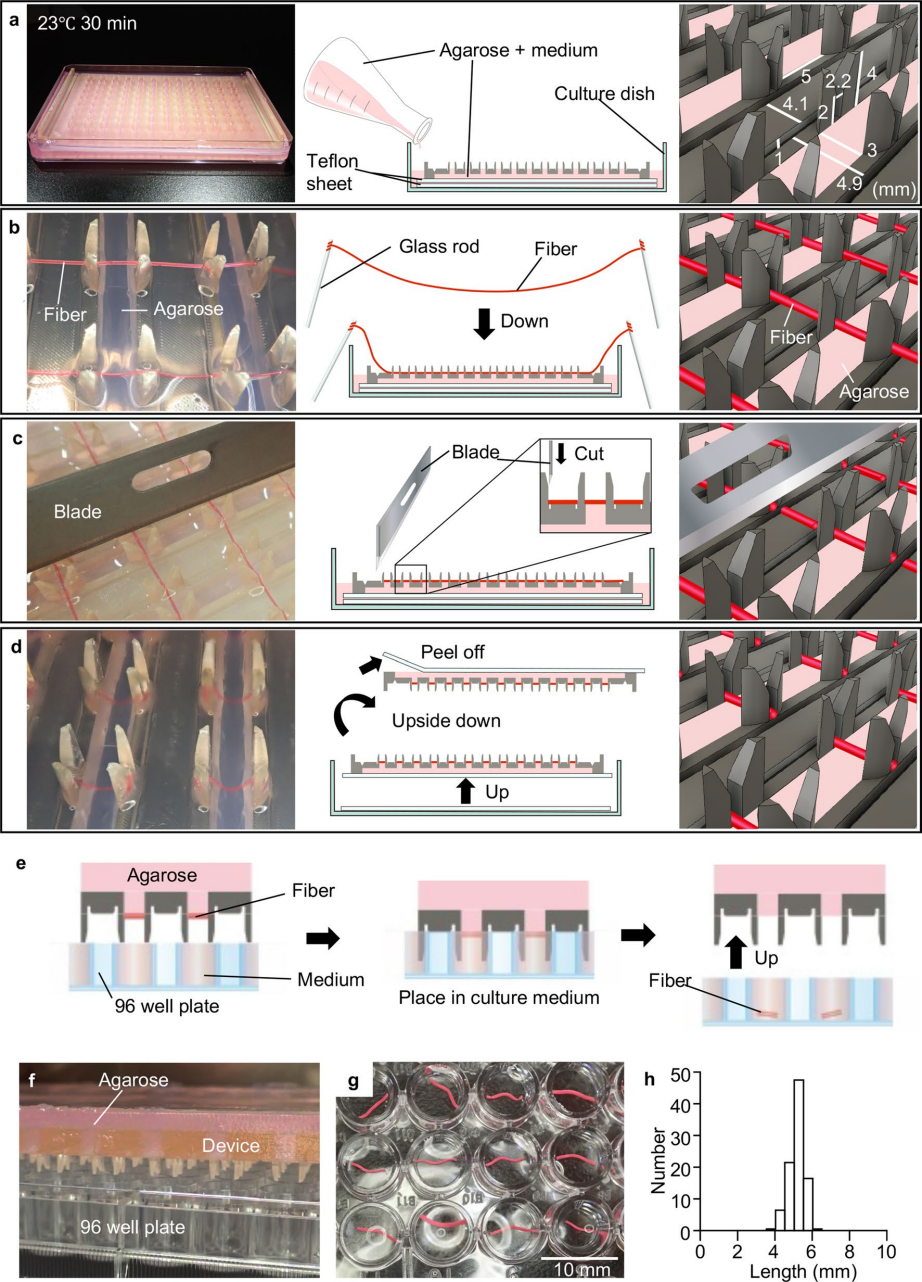
Formation of an array of the CF fragments on a 96-well plate. (**a**) Photograph of the fabricated device (left), illustration of device fabrication (center), and enlarged illustration of the device (right). (**b**) Photograph of the fiber installation on the device (left), illustration of the fiber installation on the device (center), and enlarged illustration (right). (**c**) Photograph of the cutting fibers on the device with a blade (left), illustration of the cutting fibers on the device with a blade (center), and enlarged illustration (right). (**d**) Photograph (left), illustration (center), and enlarged illustration (right) of the device with the fibers removed from the square dish. (**e**) Illustration of the transfer of the fibers from the device to a 96-well plate, (**f**) side view of the transfer of the device to a 96-well plate. (**g**) Photograph of the fibers transferred to a 96-well plate, and (**h**) graph showing the measured length of the fibers in a 96-well plate array. The length of the fiber was 5.18 ± 0.45 mm (mean ± SD, n = 96).

To fabricate 3D tissue arrays, a cell fiber is cut into millimeter-sized sections on our developed fiber-cut-and-transfer (FCAT) device and is transferred onto a 96-well plate. The FCAT device comprises agarose gel and frame fabricated by a 3D printer (Fig. 2a). The cell fibers are affixed to the FCAT device using two glass rods (Fig. 2b) and are then sectioned (Fig. 2c). The device is turned over (Fig. 2d) and gently placed on a 96-well plate (Fig. 2e, f). The fiber fragments are transferred onto a 96-well plate (Fig. 2g), thus completing the CF fragment array. Using the array of the CF fragments, we evaluate the reactive oxygen species (ROS)-induced cytotoxicity in cell lines, cancer cells, neural stem cells, and neurons, and the effectiveness of this system in drug screening. Furthermore, we clarify the differences in ROS sensitivity among different cell types and in the proliferative and trophic effects of β-estradiol. We also examined the effects of the anticancer drug, doxorubicin (DOX), using the CF fragments formed by HeLa cells. We demonstrate that the array of CF fragments is a system that can evaluate cytotoxicity and cell proliferation.

## Results

### Fabrication of the CF fragment array on a 96-well plate

To prepare an array of the cell fiber fragment with uniform shape and length, we developed an FCAT device that can fix eight cell fibers with lengths in the range of 15–25 cm, section them in uniform sizes using a disposable microtome blade, and transfer them to a 96-well plate (Fig. 2). The FCAT device was made of a biocompatible resin frame and agarose, which contained cell culture media. The agarose not only keeps the device moist so that the cells do not dry out, but also allows them to affix the cell fiber. The agarose sites also help the fibers to peel off when dipped in a medium during transfer (Supplementary Movies 1 and 2).

We prepared red-ink-containing fibers to determine whether CF fragments of uniform length were sectioned and transferred to the 96-well plate (Fig. 2f,  g) and evaluated the length of the sectioned and transferred fibers. More than 90% of the fiber fragments were transferred to the 96-well plate by placing the FCAT device on the 96-well plate in a manner such that the fiber fragments were in firm contact with the liquid surface of the plate. Subsequently, all the remaining fibers were transferred to the plate by shaking the FCAT device and the 96-well plate tightly together (Supplementary Movie 1). Using the FCAT device, we successfully arrayed 5.18 ± 0.45 mm fiber fragments (mean ± standard deviation (SD), coefficient of variation (CV) (%) = 8.7%, n = 96) fiber fragments in a 96-well plate (Fig. 2h). The transfer rate of fragment fibers was 100%, and the CV value of their lengths was less than 10%. These results indicate that the FCAT device could easily prepare fiber fragments with uniform length and array them following their transfer to a 96-well plate.

### Characteristics of the array of CF fragments compared to the array of spheroids

**Figure 3:**
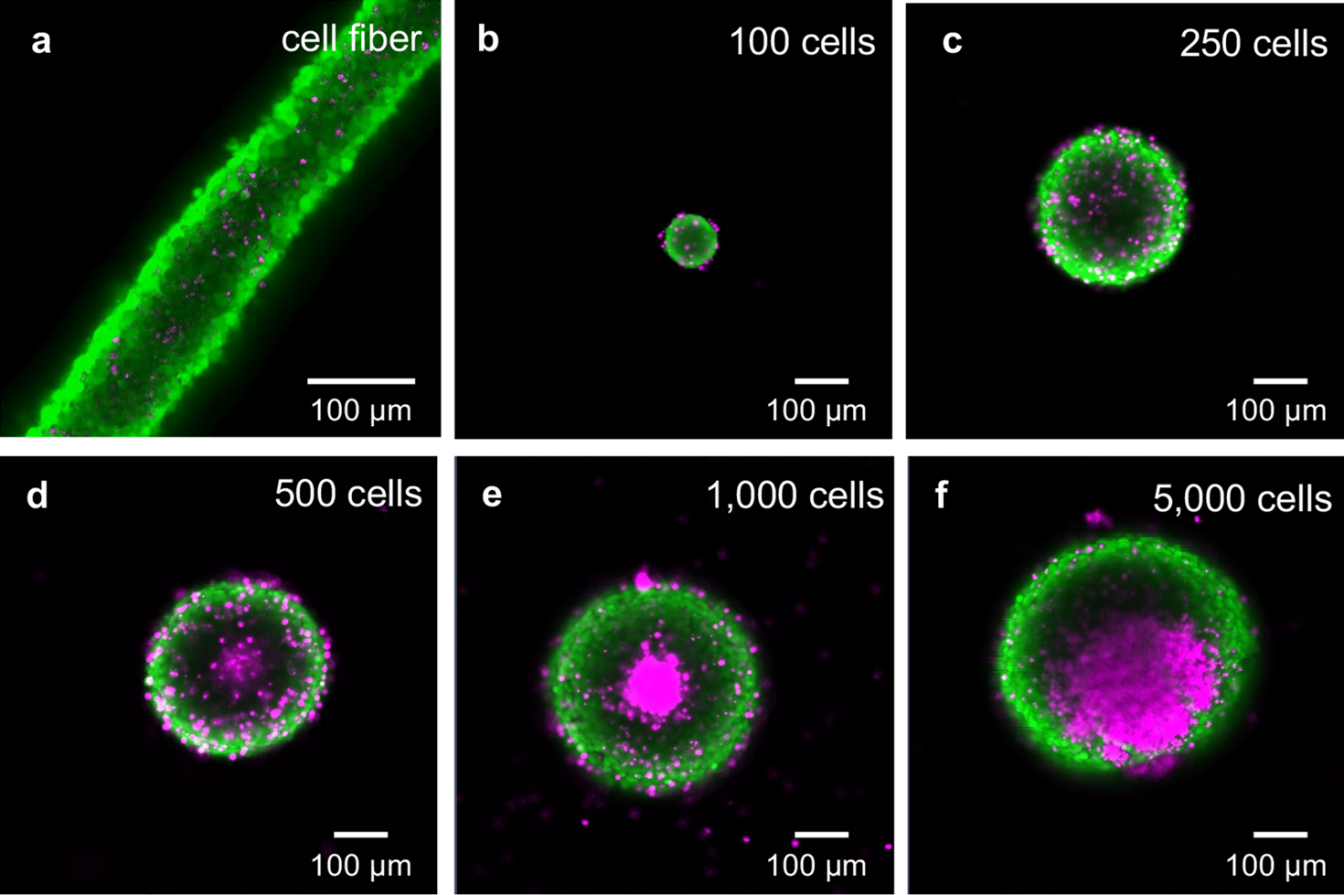
Live/dead cell viability assay of GT1-7 CF fragment and GT1-7 spheroids. GT1-7 CF fragments and GT1-7 spheroids fabricated with different initial cell numbers (100, 250, 500, 1000, and 5000 cells) were cultured for 11 days. The cells were stained with the live/dead cell staining kit II. (**a**) Fluorescence image of live/dead assay of GT1-7 CF fragment. (**b**)–(**f**) Fluorescence images of live/dead assay of GT1-7 spheroids fabricated with different initial cell numbers (100, 250, 500, 1000, and 5000 cells). The live and dead cells exhibited green and magenta fluorescence, respectively.

**Figure 4:**
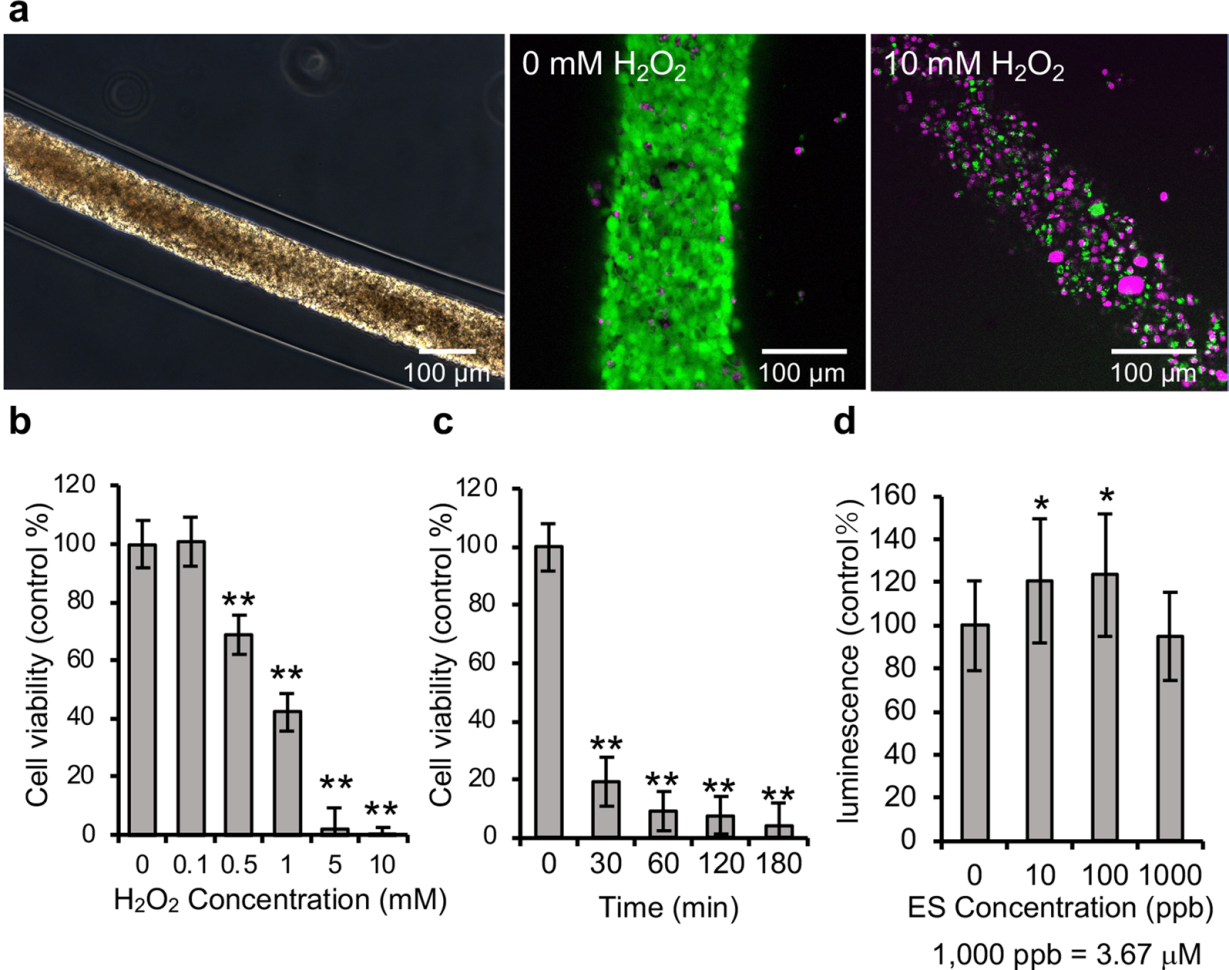
Cell viability of GT1-7 CF fragments following H_2_O_2_ and β-estradiol treatment. (**a**) Phase-contrast image of the GT1-7 CF fragment at 10 days in culture (left). Fluorescence images of live/dead assay of GT1-7 CF fragments treated with 0 or 10 mM H_2_O_2_ for 24 h after 10 days in culture (middle and right). The live and dead cells exhibited green and magenta fluorescence, respectively. (**b**) Graphs of cell viability after treatment of GT1-7 CF fragments at 10 days in culture with various H_2_O_2_ concentrations (0–10 mM) for 24 h, (**c**) with 5 mM H_2_O_2_ for 30, 60, 120, and 180 min. (**d**) Graph of the luminescence after treatment of GT1-7 CF fragments at 10 days in culture with 0–1000 ppb β-estradiol (ES) for 48 h. The experiment was repeated three times with n = 4 (** *P* < 0.01, * *P* < 0.1).

To investigate whether the arrays of CF fragments have characteristics of 3D tissue that can be used for drug screening, we evaluated the cell death and cell number, and compared them to the arrays of spheroids made on 96-well plates. For this experiment, we used mouse hypothalamic GnRH neurons (GT1-7) that have been used in drug assays^23–27^. To maintain the same culture conditions, the CF fragments and the spheroids were cultured for the same number of days. In the CF fragments and small spheroids made with an initial cell number ≤ 250, no cell death was observed in the center of tissues (Fig. 3a–c). By contrast, cell death was observed in the center of large spheroids constructed with the initial cell number ≥ 500 (Fig. 3d–f). The cell number of a CF fragment was 1.2 ± 0.1 × 10^5^ (mean ± SD) cells, and the variation of the cell number was small (CV = 9.9%) (Table 1). The cell number of GT1-7 spheroids increased as a function of the initial cell number (Supplemental Fig. 1). The cell number of spheroids with an initial cell number of 250 was 1.4 ± 1.1 × 10^3^ (mean ± SD). The variation of cell number was 76.7%. Given that the bioluminescence assay is extensively used in tissues with cell numbers > 10^3^ cells^7,28–30^, the array of CF fragments had a sufficient number of cells for the bioluminescence assay. Furthermore, the array of small spheroids exhibited a large variation in cell number (CV > 30%); and there were spheroids with cell numbers < 10^3^. CF fragments also maintained a uniform shape at the time of the experiment because they were covered with an alginate shell until the instants before the experiments (Fig. 4a). Small spheroids had nonspherical shapes (Supplemental Fig. 2). These results indicate that arrays of CF fragments (compared with spheroids) exhibit the characteristics of 3D tissue arrays that are suitable for drug screening, no cell death is observed in the center of the tissue, uniform cell number, and sufficient cells for the assay and uniform shape.

**Table 1:** Cell numbers of GT1-7 CF fragment and GT1-7 spheroids.

	CF fragment	Spheroid (initial cell number)				
		50	100	150	200	250
Cell number (mean ± SD, n = 20)	1.2 ± 0.1 × 10^5^	184 ± 86	259 ± 132	314 ± 125	689 ± 273	1420 ± 1090
(CV (%))	(9.9)	(46.8)	(51.1)	(39.7)	(39.6)	(76.7)

GT1-7 CF fragments and GT1-7 spheroids made with the different initial cell numbers (50, 100, 150, 200, 250 cells) were cultured for 11 days. The numbers of cells in CF fragments and spheroids were measured.

### Drug response assay using the array of CF fragments

To compare the assay quality of the arrays of CF fragments and small spheroids, we prepared the arrays of CF fragments and small spheroids from GT1-7 cells and performed drug response assays to H_2_O_2_ that induced cell death^26,31^. In the array of the CF fragments, H_2_O_2_ treatment for 24 h induced cell death in a concentration-dependent manner (Fig. 4a, b). There was no change in cell viability at 0.1 mM H_2_O_2_, while the quantified cell viability at 10 mM H_2_O_2_ was 0.14 ± 0.04% (mean ± SD). The half-maximal effective concentration (EC50) of H_2_O_2_ was 0.84 mM. In the array of small spheroids (with the initial cell numbers of 250 and 200), there was a large variation in the measurement data at 0 mM (Supplemental Fig. 2). In the bioluminescence assay, the sensitivity (signal-to-background ratio (S/B)) of the assay system using the GT1-7 CF fragments was > 1000, and the S/B ratio of small spheroids was < 100, because the mean signal increases when the cell number is large. We also calculated the z’-factor, which is a measure of assay quality^32,33^ (Table 2). In three independent measurements, the z’-factor was 0.7, 0.8, and 0.9 for the array of CF fragments, respectively (Supplemental Fig. 3). The z’-factor was − 3.6, − 2.1, and − 0.7 for the arrays of spheroids with initial cell numbers equal to 250, and − 2.3, − 2.1, − 0.7 for the arrays of spheroids with initial cell number equal to 200 (Supplemental Fig. 3). The array of CF fragments passed the acceptance criteria for drug screening (z’-factor ≥ 0.5)^33^, but the array of small spheroids did not. These results suggest that the array of CF fragments had higher sensitivity and assay quality than the array of small spheroids in the bioluminescence assay.

**Table 2:** Z’-factors of arrays of the CF fragments on a 96-well plate.

	GT1-7 fiber	HeLa fiber	mNSC fiber
Z’-factor	0.8 (H_2_O_2_)	0.5 (H_2_O_2_) 0.7 (DOX)	0.5 (H_2_O_2_)

The z’-factors obtained from bioluminescence assays for cell viability assessments using the arrays of the CF fragments of GT1-7 cells, HeLa cells, and mNSCs were calculated. The z’-factors were calculated by substituting the values for the positive and negative control in the Eq. (2). The acceptance criterion was set as follows: z’-factor ≥ 0.5.

We examined the response of GT1-7 CF fragments to short-term treatments of H_2_O_2_ and to β-estradiol with cell proliferative and trophic effects to determine whether arrays of GT1-7 CF fragments were able to evaluate the responses to different drug treatment times and to different drugs^34,35^ (Fig. 4c, d). Following short-term treatments with 5 mM H_2_O_2_, the cell viability of the 30 min treatment was 19.1 ± 8.9% (mean ± SD). Following a 180 min treatment, the cell viability was 4.3 ± 3.1% (mean ± SD), which was close to the cell viability obtained following 24 h treatments, 1.9 ± 2.6% (mean ± SD). With β-estradiol treatment of the array of GT1-7 CF fragments for 48 h, the amount of ATP detected as luminescence increased, which was 120.7 ± 28.5% (mean ± SD) versus the control at 10 parts per billion (ppb) β-estradiol (36.7 nM) and 123.4 ± 28.3% (mean ± SD) at 100 ppb β-estradiol (367 nM) (Fig. 4d). The amount of ATP detected as luminescence returned to 94.8 ± 20.5% (mean ± SD) after treatment with a β-estradiol concentration of 1000 ppb (3.67 μM). The increase in ATP amounts suggests that the cells were proliferating. These results indicate that arrays of GT1-7 CF fragments can evaluate differences in the treatment time of drugs and can also evaluate robustly cell proliferative and trophic effects for cells such as β-estradiol.

**Figure 5:**
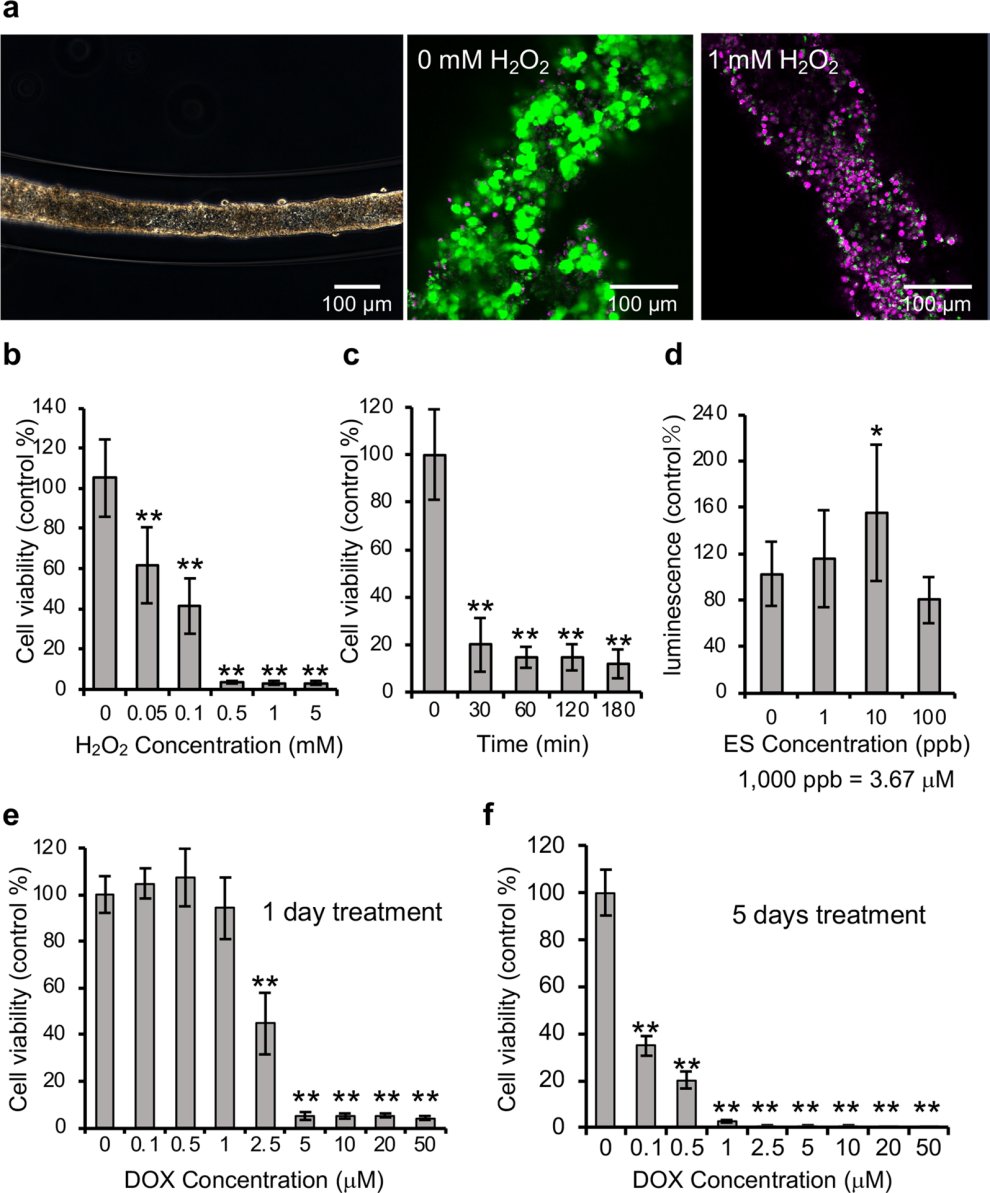
Cell viability of HeLa CF fragments following H_2_O_2_, β-estradiol, and DOX treatment. (**a**) Phase-contrast image of HeLa CF fragment at 10 days in culture (left). Fluorescence images of live/dead assay of HeLa CF fragments treated with 0 or 1 mM H_2_O_2_ for 24 h after 10 days in culture (middle and right). The live and dead cells exhibited green and magenta fluorescence, respectively. (**b**) Graphs of cell viability after treatment of HeLa CF fragments at 10 days in culture with various H_2_O_2_ concentrations (0–5 mM) for 24 h, (**c**) with 0.5 mM H_2_O_2_ for 30, 60, 120, and 180 min. (**d**) Graph of luminescence after treatment of HeLa CF fragments at 10 days in culture with 0–100 ppb β-estradiol (ES) for 48 h. (**e**) Graphs of cell viability after treatment of HeLa CF fragments at 10 days in culture with various DOX concentrations (0–50 μM) for 1 day and (**f**) 5 days. The experiment was repeated three times with n = 4 (** *P* < 0.01, * *P* < 0.1).

**Figure 6:**
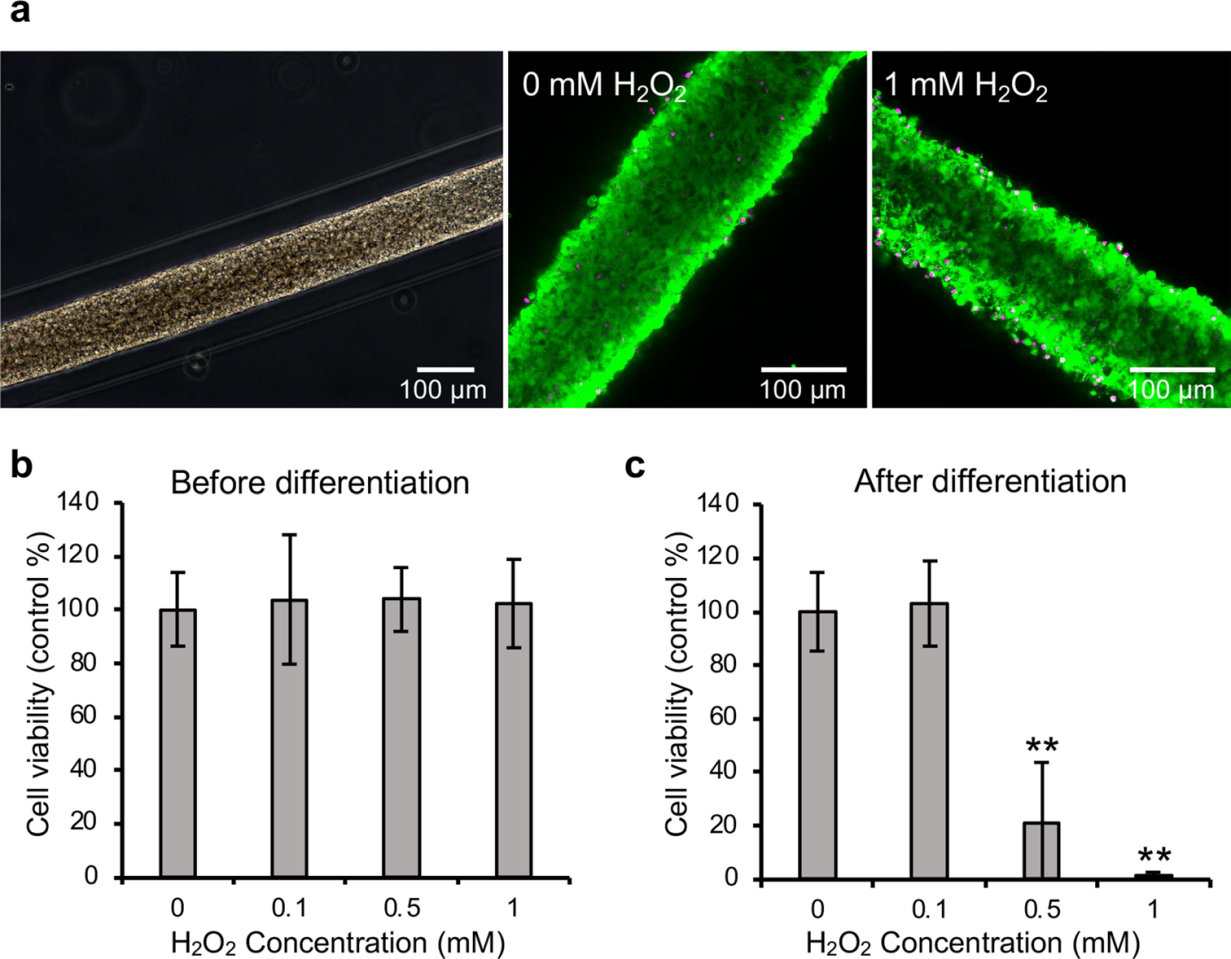
Cell viability of mNSC CF fragments before or after differentiation following H_2_O_2_ treatment. (**a**) Phase-contrast image of mNSC CF fragment at 10 days in culture (left). Fluorescence images of live/dead assay of mNSC CF fragments treated with 0 or 1 mM H_2_O_2_ for 24 h after 10 days in culture (middle and right). The live and dead cells exhibited green and magenta fluorescence, respectively. (**b**) Graph of cell viability after treatment of mNSC CF fragments at 10 days in culture with various H_2_O_2_ concentrations (0–1 mM) for 24 h. (**c**) Graph of cell viability after treatment of mNSC CF fragments at 10 days in culture after differentiation with various H_2_O_2_ concentrations (0–1 mM) for 24 h. The experiment was repeated three times with n = 4 (** *P* < 0.01).

Additionally, to confirm the versatility of the array of CF fragments in drug screening assays, we fabricated arrays of CF fragments from cancer cells (HeLa), mouse neural stem cells (mNSCs), and differentiated mNSCs and performed drug response assays to H_2_O_2_, β-estradiol, and DOX. In the array of the HeLa CF fragments, the 24 h H_2_O_2_ treatment induced cell death in a concentration-dependent manner similar to the GT1-7 CF fragments (Fig. 5a, b). The cell viability was 39.9 ± 14.8% (mean ± SD) at 0.1 mM H_2_O_2_ and 3.4 ± 0.5% (mean ± SD) at 0.5 mM H_2_O_2_. The EC50 was 85 μM. The z’-factor was 0.5 (Table 2). In the short-term treatment of 0.5 mM H_2_O_2_, the cell viability of the 30 min treatment was 20.0 ± 11.3% (mean ± SD) (Fig. 5c). At 180 min treatment, the cell viability was 11.8 ± 6.1% (mean ± SD), which was close to the cell viability obtained following a 24 h treatment, i.e., 3.4 ± 0.5% (mean ± SD). The β-estradiol treatment to the array of HeLa CF fragments for 48 h resulted in an increase in cell viability of 155.3 ± 58.6% (mean ± SD) at 10 ppb (36.7 nM) (Fig. 5d). The cell viability decreased to 80.0 ± 19.4% (mean ± SD) following a β-estradiol treatment at 100 ppb (367 nM). DOX treatment for 24 h induced cell death in a concentration-dependent manner (Fig. 5e). The cell viability was 94.3 ± 13.1% and 5.2 ± 1.9% (mean ± SD) following treatment with 1 μM DOX and 5 μM DOX, respectively. The half-maximal inhibitory concentration (IC50) of DOX was 2.4 μM. After 5 days of treatment with DOX, the cell viability after 0.1 μM DOX treatment was 35.0 ± 4.3% (mean ± SD) (Fig. 5f). The z’-factor was 0.7 and 0.7 after treatment for 1 day and 5 days, respectively. In the array of spheroids with initial cell numbers of 100, the diameter of the spheroids was > 200 μm, and a large variation was observed in the measurement data at 0 mM (CV = 33%, n = 36, Supplemental Fig. 4). In the array of spheroids with an initial cell number of 500, the CV was 29% at 0 mM (n = 36, Supplemental Fig. 4). The z’-factor was 0 and − 0.1 for the arrays of spheroids with initial cell numbers equal to 100 and 500, respectively. After 24 h of H_2_O_2_ treatment of the CF fragment array of mNSCs, the cell viability remained intact for H_2_O_2_ concentrations in the range of 0–1 mM (Fig. 6a, b). Therefore, we also treated the array of CF fragments of differentiated mNSCs with H_2_O_2_ for 24 h. Cell death was induced in a concentration-dependent manner similar to the GT1-7 and HeLa cells (Fig. 6c). The cell viability was 21.3 ± 22.5% (mean ± SD) after 0.5 mM H_2_O_2_ treatment and 1.5 ± 0.9% (mean ± SD) after 1 mM H_2_O treatment. The EC50 was 0.47 mM, and the z’-factor was 0.5 (Table 2). These results suggest that arrays of CF fragments made from other cell types can also pass the acceptance criteria for drug screening (z’-factor ≥ 0.5)^33^, and that these arrays of CF fragments can be used for the evaluation of cell death and cell proliferation.

## Discussion

In this study, we developed an FCAT device that fixed cell fibers, sectioned them into uniform sizes, and transferred them to a 96-well plate to fabricate 3D tissue arrays for drug screening. Using the FCAT device, we successfully fabricated an array of CF fragments with uniform shape and size and no internal cell death. The GT1-7 CF fragment contained 1.2 ± 0.1 × 10^5^ cells, and the variabilities of both cell number and cell viability assays were small. Using a variety of cell types (cell lines, cancer cells, neural stem cells), we fabricated the arrays of CF fragments and detected differences in reactivity to H_2_O_2_ and β-estradiol depending on the cell type and based on whether differentiation was induced. Z’-factors of an assay performance measurement passed the acceptance criteria (z’-factor ≥ 0.5) for all CF fragments, indicating that our assays using CF fragments can be recognized as an excellent assay^33^.

The shape of the tissue in the array of CF fragments was more uniform than that in the array of spheroids. Given that spheroids allow cells to grow in any direction, the uniformity of the shape differs dramatically depending on the cell type^7,16,36^. Moreover, the variation in the cell number by pipetting at low-seeding densities prevents the production of uniform-sized spheroids^17^. By contrast, CF fragments are made by sectioned tube-shaped tissues that are uniformly filled with cells and covered with alginate hydrogel to the same length with the FCAT device. Therefore, even if the cells do not form spheroids of uniform shapes, such as GT1-7, we can obtain 3D tissues of uniform shape by preparing CF fragments.

The array of CF fragments reproducibly detected drug responses that changed with cell type and between the 2D culture system and 3D tissues. We could robustly detect these responses using the array of CF fragments such that neural stem cells resistant to ROS became more sensitive to ROS after differentiation induction^37,38^. Furthermore, the array of CF fragments allowed us to detect the H_2_O_2_ reactivity specific to 3D tissues; that is, the cell death-inducing effect of H_2_O_2_ was lower in CF fragments than in 2D cultures (GT1-7; EC50 of 2D culture = 0.3 mM (Supplemental Fig. 5), EC50 of CF fragments = 0.84 mM, HeLa; EC50 of 2D culture = 75 μM^39^, EC50 of CF fragment = 85 μM). This is because H_2_O_2_ acts on all cells at once in 2D culture, while in 3D tissue, only cells on the tissue surface are affected by H_2_O_2_^40^, suggesting that the EC50 is higher in 3D tissue. In 2D cultures, β-estradiol has been reported to have proliferative and trophic effects at low concentrations, but these effects are suppressed at high concentrations^34,35^. We were able to detect this effect reproducibly using CF fragment arrays. We also found a difference in the effect of the anticancer drug, DOX, between 2D culture systems and 3D tissues of HeLa cells. CF fragments showed lower drug sensitivity to DOX than 2D culture, but higher sensitivity than spheroids (HeLa; IC50 of 2D culture = 0.7–1.45 μM^41–43^, IC50 of spheroid = 10 μM (3 days treatment of DOX)^43^, IC50 of CF fragments = 2.4 μM). It has been reported that spheroids covered with matrix gels are more sensitive to DOX than floating spheroids because the gels accumulate the drug^44^. In CF fragments, the higher drug sensitivity than spheroids may be due to the collagen gel contained in CF fragments. The higher drug sensitivity than the spheroid is thought to be an effect of the collagen gel in the CF fragment. Recently, the effect of the cellular matrix to anticancer drugs in cancer tissues has also attracted attention^44–46^, and the experimental data using CF fragments containing collagen gel will be an important indicator to observe the effect of the cellular matrix. By using CF fragment arrays, we can provide an assay that can detect 3D tissue-specific drug responsiveness close to that observed in vivo and changes in drug responsiveness to dynamically changing cells, such as differentiation induction.

The array of CF fragments was a 3D tissue array without cell death in the center of the tissue regardless of cell type. Conventional spheroid assays have been used specifically for screening of anticancer drugs using arrays of large spheroids that require cell death in the center of the tissue, such as in vivo cancer tissues^7,12,17^. Recent studies have reported that organoid arrays can achieve sufficient assay quality with z’-factors exceeding 0.5 for screening of anticancer drugs^47^. 3D tissues without cell death in the center of the tissue are required to perform drug screening with non-cancer cells. In the present study, arrays of CF fragments without cell death inside the tissue were able to yield z’-factors ≥ 0.5 in the evaluation of quality and robustness of the assay. In the near future, by calculating the z’-factor of other drugs in more cells as well, the array of CF fragments without internal cell death could be extensively used for drug screening. Moreover, using the FCAT device, we can create uniform and standardized 3D tissues of various cell types, and quantitatively analyze changes in gene expression and protein levels in tissues, as well as perform simple cell viability assay.

## Methods

### Fabrication of the FCAT device

The FCAT device comprised a surgical guide resin (Formlabs, Somerville, MA, USA) frame, fabricated by a Formlabs Form 3B printer (Formlabs), and 3% (w/v) agarose (Lonza, Tokyo, Japan). Before the fabrication of the device, the frame and square culture dish (Asone, Osaka, Japan) and NAFLON(R) tape (polytetrafluoroethylene) (Asone) were exposed to ultraviolet light and ozone gas using a sterilizer machine (CoolCLAVE Plus, Genlantis Inc., San Diego, CA, USA). The FCAT device was prepared by setting a surgical guide resin frame on two NAFLON(R) tapes in a square culture dish. Subsequently, 3% (w/v) agarose dissolved in the medium was poured in the square culture dish and placed still for 30 min at 23 °C (Fig. 2a).

### Cell cultures

Dr. R. Weiner (University of California, San Francisco) kindly provided GT1-7 cells. GT1-7 cells were maintained using Dulbecco’s Modified Eagle’s Medium/Ham’s Nutrient Mixture F-12 (DMEM/F12, Fujifilm Wako Pure Chemical Corporation, Osaka, Japan), containing 10% (v/v) fetal bovine serum (FBS, Corning, NY, USA), and 1% (v/v) penicillin–streptomycin (Thermo Fisher Scientific K.K., Tokyo, Japan). The human cervical cancer HeLa cells (VectorBuilder Japan, Kanagawa, Japan) were maintained using Dulbecco’s Modified Eagle Medium (DMEM, Fujifilm Wako Pure Chemical Corporation), containing 10% (v/v) FBS and 1% (v/v) penicillin–streptomycin (Merck Japan, Tokyo, Japan). The mNSCs were dissected from the midbrains of ICR mice (embryonic days: 13.5) as reported previously^20–22^. ICR mice were obtained from Sankyo Labo Service Corporation Inc. (Tokyo, Japan). All experiments with mice were approved by the Animal Experiment Ethics Committee of Institute of Industrial Science in The University of Tokyo (approval no. 30–12). Experiments were performed by the guidelines by Life Science Research Ethics and Safety Committee of The University of Tokyo. All experiments were performed in accordance with the ARRIVE guidelines. The mNSCs were cultured as neurospheres in the Neurobasal-A (Thermo Fisher Scientific K.K) growth medium supplemented with 2 mM l-glutamine (Thermo Fisher Scientific K.K), 1% penicillin–streptomycin solution, 20 ng/ml basic fibroblast growth factor (bFGF, Peprotech, Rocky Hill, NJ, USA), 20 ng/ml human epidermal growth factor (hEGF, Peprotech), and B27 without vitamin A (12587-010, Thermo Fisher Scientific K.K). The mNSCs were passaged 2–3 times using TrypLE Select (Thermo Fisher Scientific) before use. All cells were maintained in a water-saturated 5% CO_2_ atmosphere at 37 °C.

### Spheroid generation and CF formation

For spheroid generation, the GT1-7 cells were seeded at initial cell concentrations of 50, 100, 150, 200, 250, 500, 1000 and 5000 cells per well at V-bottom 96-well plates (Corning). The medium was replaced by a half volume every 2–3 days. The spheroids were maintained in a water-saturated 5% CO_2_ atmosphere at 37 °C.

The cell-encapsulating core–shell hydrogel microfibers were formed using a double coaxial laminar flow microfluidic device as reported previously^20–22^. For cell fibers from GT1-7 cells and HeLa cells, we prepared three materials: (1) GT1-7 cell suspension at 1.0 × 10^8^ cells/ml in 2 mg/ml pepsin-solubilized type-I collagen (PCol), neutralized in DMEM (AteloCell™, DME-02, KOKEN, Toyko, Japan) for the core stream, and HeLa cell suspension at 1.0 × 10^8^ cell/ml in 2 mg/ml PCol, neutralized in DMEM for the core stream; (2) 1.5% v/v sodium alginate (194-13321, Fujifilm Wako Pure Chemical Corporation) in 145 mM sodium chloride (NaCl, 191-01665, Fujifilm Wako Pure Chemical Corporation) solution for the shell stream, and (3) 100 mM calcium chloride (CaCl_2_, 7057-00, Kanto Chemical Co., Inc., Tokyo, Japan) and 3% w/w sucrose (304355, Nacalai Tesque Inc., Kyoto, Japan) solution for the sheath stream. For cell fibers from mNSCs, we prepared three materials: (1) mNSC suspension at 2.0 × 10^8^ cell/ml in 2 mg/ml PCol, neutralized in DMEM with 100 ng/ml bFGF and 100 ng/ml hEGF for the core solution stream, (2) 1.5% v/v sodium alginate (194-13321, Fujifilm Wako Pure Chemical Corporation) in 145 mM sodium chloride (NaCl, 191-01665, Fujifilm Wako Pure Chemical Corporation) solution for the shell stream, and (3) 100 mM calcium chloride (CaCl_2_, 7057-00, Kanto Chemical Co., Inc., Tokyo, Japan.) and 3% w/w sucrose (304355, Nacalai Tesque Inc.) solution for the sheath stream. The flow rates of each stream—core, shell, and sheath—were *Q*_core_ = 25 μl/min, *Q*_shell_ = 75 μl/min, and *Q*_sheath_ = 3.6 ml/min, respectively. The continually formed cell-encapsulating core–shell hydrogel fibers were collected in a sterile 145 mM NaCl solution in a tube. These cell fibers were then cultured in the respective media. All cells were maintained in a water-saturated 5% CO_2_ atmosphere at 37 °C. For the cell fibers of GT1-7 cells and HeLa cells, the medium was changed every 2–3 days. For the mNSC fiber, 20 ng/ml bFGF and 20 ng/ml hEGF were added every 2–3 days.

### Fabrication of an array of CF fragment on a 96-well plate

The agarose on the device was moistened with a small amount of medium to prevent it from drying out before use. The 96-well plate was filled with 300 μl/well of medium and prepared for transfer. The fibers of GT1-7 cells, HeLa cells, and mNSCs were cultured for 9 days. Some mNSC fibers were replaced with differentiation induction medium (Neurobasal-A medium supplemented with 2 mM L-glutamine, 1% penicillin–streptomycin solution, and B27 with vitamin A (Thermo Fisher Scientific K.K) and cultured for 10 days. The cell fiber of GT1-7 cells, HeLa cells, or mNSCs (differentiated fiber, undifferentiated fiber) were affixed to the FCAT device using two glass rods (Fig. 2b) and then sectioned using a disposable microtome blade (Feather) along the guide (Fig. 2c). Unwanted fibers were gently removed from the device with flat tweezers. The device was removed from the square culture dish with the first NAFLON(R) Tape and turned over (Fig. 2d). The NAFLON(R) tape was removed, and the device was gently placed on a 96-well plate (Fig. 2e, f). The 96-well plate along with the device was shaken up and down to ensure that all the fiber fragments were transferred (Supplementary Movies 1 and 2). The device was removed from the 96-well plate, and 100 µl of medium was removed from each well and incubated in a water-saturated 5% CO_2_ atmosphere at 37 °C.

Fibers filled with water-based ink (POSCA, Mitsubishi Pencil, Tokyo, Japan) were prepared instead of cells to evaluate the transfer rate of the device and uniformity of the fiber fragments. The transfer rate was calculated by measuring the number of fibers transferred to a 96-well plate. Images of the transferred fiber fragments obtained using a Biotek Cytation 5 plate reader (Biotek Instruments, City, VT, USA) and stereomicroscope (Lica Microsystems) were quantified using the default plugin in ImageJ (version 1.51, National Institutes of Health, Bethesda, MD, USA) to evaluate the uniformity of the fiber fragments.

### Cell counting

The CF fragments of the 96-well plate array were treated with 80 µg/ml arginase for 5 min at 37 °C to remove the alginate hydrogel. The CF fragments were then washed twice with Dulbecco’s phosphate-buffered saline (DPBS) (Merck Japan). The spheroids were washed once with DPBS. The CF fragments and spheroids were treated with CTS™ TrypLE™ Select Enzyme (Thermo Fisher Scientific K.K) for 5–20 min at 37 °C and cell counts were measured.

### Cell viability assays

To visualize live and dead cells, a live/dead cell staining kit II (Takara, Kyoto, Japan) was used on spheroids and CF fragments. Fluorescence was detected using a confocal laser microscope (LSM710, Carl Zeiss). A cell viability assay was performed on spheroids and CF fragments using CellTiter-Glo3D (Promega Corporation, Madison, WI, USA) according to the manufacturer’s instructions. The viability of the spheroids or CF fragments was measured by reading the luminescence. CellTiter-Glo3D reagent (100 µL/well) was added, placed on a shaker for 5 min, and then equilibrated at room temperature for 25 min to stabilize the luminescence signal. The luminescence was then measured using a Biotek Cytation 5 plate reader (Biotek Instruments, VT, USA). The data were obtained in quadruple and expressed as mean ± SD of viable cells relative to the control. The cell viability assay was repeated three times.

### Drug treatments

Drug screening was performed using CF fragments of 96-well plate arrays treated with 80 μg/ml of arginase for 5 min at 37 °C to remove the alginate hydrogel. They were then washed twice with culture media. The CF fragments were treated with H_2_O_2_ (Nakarai Tesque, Kyoto, Japan) or doxorubicin hydrochloride (Fujifilm Wako Pure Chemical Corporation) for 24 h or for shorter time intervals (15, 30, 60, 120, 180 min); a live/dead cell staining kit II or CellTiter-Glo3D was used. β-estradiol (Merck Japan) was prepared on 1 mg/ml with dimethyl sulfoxide. For β-estradiol treatment, cell fibers were cultured for 9 days, then washed twice with serum-free media and cultured overnight in serum-free media. Subsequently, CF fragments in 96-well plate arrays were made from these fibers and treated with 80 μg/ml arginase for 5 min at 37 °C to remove the alginate hydrogel. The CF fragments were treated with β-estradiol for 48 h. The cell viability was estimated by CellTiter-Glo3D.

### Assay validation

The CV was calculated as follows:

1 $$CV\left( \% \right) = \frac{SD}{{AV}} \times 100$$

The assay system of the array of CF fragments on a 96-well plate was evaluated based on the z’-factor. The z’-factor is an assay performance measurement, which easily summarizes the assay’s quality and robustness^17,33,48^. The range of z’-factor ranges from negative infinity to one, with values ≥ 0.5 indicating an excellent assay, > 0 an acceptable assay (double assay), 0 = yes/no type assay, and < 0 an unacceptable assay^17,33^. Z’-factors, commonly reported in assay evaluation, were calculated by substituting the values for positive and negative control in the following Eq. (2).

2 $$\begin{gathered} Z' = 1 - \frac{{3 \times \left( {SD_{100} + SD_{0} } \right)}}{{AV_{100} - AV_{0} }}\,\,\,\,\,\,SD;\,\,{\text{standard}}\,\,{\text{deviation}} \hfill \\ \,\,\,\,\,\,\,\,\,\,\,\,\,\,\,\,\,\,\,\,\,\,\,\,\,\,\,\,\,\,\,\,\,\,\,\,\,\,\,\,\,\,\,\,\,\,\,\,\,\,\,\,\,\,\,\,\,AV;\,\,{\text{average}} \hfill \\ \end{gathered}$$

Acceptance criteria were set at z' factors ≥ 0.5 and CV values (%) < 10%.

### Statistical analysis

The statistical analyses were performed using Microsoft Excel and Igor Pro 8.04. All data are expressed as mean ± SD. Significant differences among each group were examined using one-way of analysis of variance. For statistical analyses, a two-tailed Student’s t test (with *P* < 0.05 being considered significant) was used for pairwise group comparisons.

## Supplementary Information

Supplementary Video 1.

Supplementary Video 2.

Supplementary Information 1.

## Data Availability

The data that support the findings in this study are available from the corresponding author upon reasonable request.
