# Supplementary material for: Cell fiber-based 3D tissue array for drug response assay
Source: Sci Rep. 2022 May 12;12:7870. doi: 10.1038/s41598-022-11670-2 (PMC9098497; doi:10.1038/s41598-022-11670-2)
Supplement: Supplementary file 3 — Supplementary Information 1. [file 41598_2022_11670_MOESM3_ESM.docx]

Supplementary Information

for *Scientific Report*

Title:

Cell fiber-based 3D tissue array for drug response assay

Midori Kato-Negishi, Jun Sawayama, Masahiro Kawahara and Shoji Takeuchi*

Contents

1. Supplemental Figure 1. Cell number of GT1-7 spheroids.
2. Supplemental Figure 2. Cell viability of GT1-7 spheroids following H_2_O_2_ treatment.
3. Supplemental Figure 3. Z’-factor of the arrays of GT1-7 spheroids and GT1-7 CF fragments on a 96-well plate.
4. Supplemental Figure 4. Cell viability of HeLa spheroids following DOX treatment.
5. Supplemental Figure 5. Cell viability of 2D-cultured GT1-7 cells.
6. Supplementary Movie 1. Top view of transfer of fiber fragments in a 96-well plate processed in the FCAT device.
7. Supplementary Movie 2. Side view of transfer of fiber fragments from FCAT device to a 96-well plate.

**1. Supplemental Figure 1. Cell number of GT1-7 spheroids.**

GT1-7 cell spheroids fabricated with different initial cell numbers (50, 100, 150, 200, 250, 500, 1000, and 5000 cells) were cultured for 11 days. The number of cells in spheroids was measured.

**2. Supplemental Figure 2.** **Cell viability of GT1-7 spheroids following H_2_O_2_ treatment.**

(a) Phase-contrast image (upper) of the GT1-7 spheroid with an initial cell number 200 cells at 11 days in culture. Cell viability (bottom) of GT1-7 spheroids with an initial cell number 200 cells. GT1-7 spheroids were cultured for 10 days and treated with various H_2_O_2_ concentrations (0–5 mM) for 24 h. Cell viability was estimated by CellTiter-Glo3D. (b) Phase-contrast image (upper) of the GT1-7 spheroids of initial cell number 250 cells at 11 days in culture. Cell viability (bottom) of the GT1-7 spheroids initial cell number 250 cells. GT1-7 spheroids were cultured for 10 days and treated with various H_2_O_2_ concentrations (0–5 mM) for 24 h. Cell viability was estimated by CellTiter-Glo3D.

**3. Supplemental Figure 3. Z’-factor of the arrays of GT1-7 spheroids and GT1-7 CF fragments on a 96-well plate.**

The z’-factors for the arrays of the GT1-7 CF fragments and GT1-7 spheroids were calculated. The acceptance criteria were set at a z’-factor ≥ 0.5.


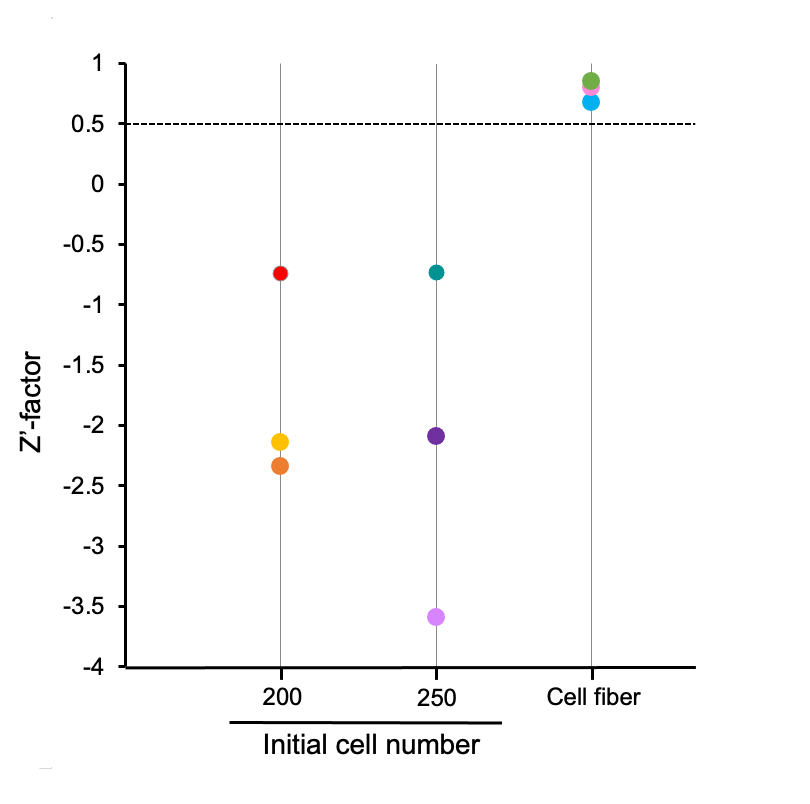


4. Supplemental Figure 4. Cell viability of HeLa spheroids following DOX treatment.

(a) Cell viability of the HeLa spheroids with an initial cell number of 100. HeLa spheroids were cultured for 10 days and treated with various DOX concentrations (0–50 μM) for 24 h. Photograph of HeLa spheroids after 0 μM DOX treatment (left). Cell viability was estimated by CellTiter-Glo3D (right). (b) Cell viability of HeLa spheroids with an initial cell number of 500. HeLa spheroids were cultured for 10 days and treated with various DOX concentrations (0–50 μM) for 24 h. Photograph of HeLa spheroids after 0 μM DOX treatment (left). Cell viability was estimated by CellTiter-Glo3D (right).


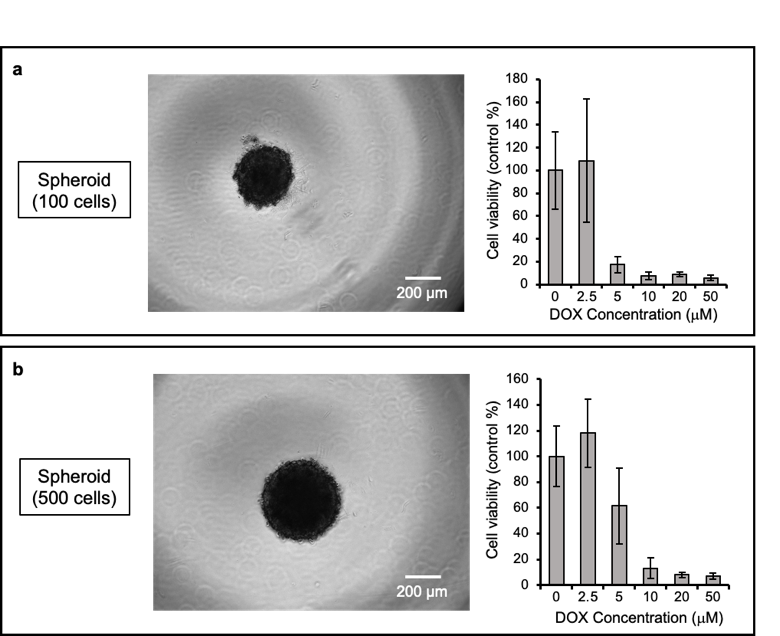


**5.** **Supplemental Figure 5.** **Cell viability of 2D-cultured GT1-7 cells.**

Dissociated GT1–7 cells were plated onto 96-well culture plates at a concentration of 5 × 10^5^ cells per well in 200 μl culture media. Following 24 h incubation, the cells were treated with various concentrations of H_2_O_2_ (0–10 mM) for 24 h. Cell viability was estimated by CellTiter-Glo 2.0　(Promega).

**6.** **Supplementary Movie 1. Top view of transfer of fiber fragments in a 96-well plate processed in the FCAT device.**

Movie of the transfer to the 96-well plate acquired from the top. The fibers were colored with water-based red ink.

7. Supplementary Movie 2. Side view of transfer of fiber fragments from FCAT device to a 96-well plate.

Movie of the transfer to the 96-well plate acquired from a side. The fibers were colored with water-based red ink.
